# Caerin 1.1/1.9 interfere KHDRBS1-DDX5 regulatory axis to induce IL-18 mediated pyroptosis in a HeLa cell tumour model

**DOI:** 10.1038/s41598-025-12450-4

**Published:** 2025-07-30

**Authors:** Mengqi Liu, Yuandong Luo, Xinyi Song, Rongmi Mo, Jiawei Fu, Quanlan Fu, Junjie Li, Jinyi Wu, Hongyin Wu, Furong Zhong, Hejie Li, Xiaosong Liu, Guoying Ni, Tianfang Wang

**Affiliations:** 1 Medical School, Guizhou University, Guiyang, 550025 Guizhou Province China; 2 Zhongao Biomedical Technology (Guangdong) Co. Ltd, Zhongshan, 528400 Guangdong China; 3 The First Affiliated Hospital, School of Clinical Medicine of Guangdong, Pharmaceutical University, Guangzhou, 510080 China; 4 Centre for Bioinnovation, University of the Sunshine Coast, Maroochydore BC, QLD 4558 Australia; 5 Cancer Research Institute, Foshan First People’s Hospital, Foshan, 528000 Guangdong China; 6 School of Science, Technology and Engineering, University of the Sunshine Coast, Maroochydore BC, QLD 4558 Australia

**Keywords:** KHDRBS1, Caerin peptide, Pyroptosis, IL-18, HeLa cell, Cervical cancer, Cancer therapy, Gynaecological cancer, Tumour immunology, Cell death and immune response, Cytokines, Immunotherapy, Inflammation, Translational immunology, Cytokines

## Abstract

Cervical cancer remains a significant global health challenge, particularly in developing countries where access to HPV vaccination is limited. We previously demonstrated that caerin 1.1/1.9 (F1F3) peptides inhibit tumour growth in vitro and in vivo by inducing pyroptosis, followed by apoptosis and immune activation. In this study, we elucidate the molecular mechanisms underlying F1F3-induced pyroptosis in HeLa cells. Our results show that F1F3 triggers pyroptosis independently of GSDME, as evidenced by comparable IL-18 and LDH release in both wild type and GSDME knockout cells. Cross-linking mass spectrometry identified the interaction of F1 to KHDRBS1 and F3 to DDX5, respectively. Knockout of either KHDRBS1 or DDX5 enhanced HeLa cell sensitivity to F1F3 and significantly elevated IL-18 secretion. Notably, KHDRBS1-deficint tumours displayed accelerated growth yet responded more robustly to F1F3 treatment, suggesting a context-dependent tumour-suppressive role of KHDRBS1. These findings uncover a previously uncharacterised pathway regulated by KHDRBS1-DDX5 and demonstrate that F1F3 can effectively interfere with this axis to induce anti-tumour immune responses, highlighting their potential as novel therapeutic agents for cervical cancer.

**Supplementary Information:**

The online version contains supplementary material available at 10.1038/s41598-025-12450-4.

## Introduction

Cancer persists as a leading cause of global mortality, with cervical cancer representing a critical burden in women’s health. Annually, over 600,000 new cases and 340,000 deaths are attributed to cervical cancer, primarily driven by persistent high-risk human papillomavirus (HPV) infections^1^. While HPV vaccination programs have reduced incidence in high-income regions, limited access to preventive care in low resource settings perpetuates disparities, with 90% of cervical cancer deaths occurring in developing nations. Advanced cervical malignancies exhibit aggressive metastasis and resistance to conventional therapies, leading to severe morbidity, including pelvic organ dysfunction and fatal hemorrhages^2^. Despite advances in immunotherapy (e.g., pembrolizumab for PD-L1^+^ tumours), 5-years survival rates for recurrent/metastatic disease remain below 20%, underscoring the imperative for novel therapeutic strategies^3^.

The limitations of current treatments, including chemoresistance, systemic toxicity, and tumour immunosuppressive microenvironments demand a paradigm shift in drug development^4^. Traditional apoptosis inducing agents often fail due to dysregulated Bcl-2 family proteins and caspase inactivation in tumours^5^. Consequently, targeting alternative programmed cell death (PCD) pathways, such as pyroptosis and necroptosis, has emerged as a breakthrough approach to circumvent resistance. Pyroptosis, a lytic and immunogenic form of programmed cell death, is characterized by gasdermin family protein-mediated membrane pore formation, followed by cytoplasmic content release (e.g., IL-1β, IL-18, and High mobility group box-1 protein) and robust activation of antitumour immunity (a dual mechanism distinct from the immunologically silent nature of classical apoptosis)^6,7^. It is primarily mediated through four pathways-: (i) the canonical pathway, involving caspase-1 activation via inflammasomes^8^; (ii) the non-canonical pathway, mediated by caspase-4/5 (human) or caspase-11 (mouse)^9^; (iii) the caspase-3-dependent pathway, where caspase-3 cleaves gasdermin E^10^; (iv) the granzyme pathway, where granzymes from cytotoxic T cells directly cleave gasdermins^11^. These pathways converge on gasdermin pore formation, leading to cell lysis and inflammation^12^. Pyroptosis is crucial in host defence and inflammatory diseases, offering therapeutic potential^13^.

Interleukin-18 (IL-18), a member of the IL-1 family of cytokines comprising 11 cytokines that collectively enhance innate immune system activity^14,15^ is a potent pro-inflammatory cytokine implicated in host defence against infections and modulation of both innate and adaptive immune responses. Produced by hematopoietic and non-hematopoietic cells, including monocytes, macrophages, keratinocytes, and mesenchymal cells, IL-18 exerts critical immunoregulatory functions through T cell activation and differentiation^16^ and upregulation of IFN-γ essential for host defence mechanisms^17^. The role of IL-18 in tumourigenesis exhibits dual characteristics, demonstrating both tumour-suppressive and tumour promoting potential. Current research indicates that IL-18 mediates antitumour effects via activation of Th1 type immune responses and enhancement of NK cell cytotoxicity^18^. Paradoxically, elevated IL-18 expression has been associated with increased tumour aggressiveness and metastatic potential in specific contexts, potentially through mechanisms involving immune escape facilitation^19^. These dichotomous effects underscore the complex therapeutic implications of IL-18 in oncology, necessitating further investigation into its context-dependent mechanisms within distinct tumour microenvironments and comprehensive evaluation of its feasibility as a therapeutic target^20,21^. IL-18 exhibits pleiotropic effects contingent upon its cytokine milieu, underscoring its pivotal pathophysiological roles in both health and disease states^22^.

KHDRBS1 (KH domain-containing RNA-binding protein 1, also known as Sam68), a member of the STAR family, is a multifunctional regulator of RNA processing, transcriptional regulation, signal transduction, and cell cycle control^23,24^. By modulating RNA splicing, stability, and translation, KHDRBS1 influences gene expression and cellular functionality^25^. Its overexpression in various cancers, such as breast, prostate, and colorectal cancers, has been linked to increased tumour aggressiveness, metastatic potential, and poor clinical prognosis^26^. Mechanistically, KHDRBS1 promotes tumour growth and metastasis through Wnt/β-catenin and PI3K/Akt signalling, as well as epithelial-mesenchymal transition induction, while inhibiting apoptosis via interactions with tumour suppressor genes such as p53^27,28^. However, its context-dependent roles in cancer require further investigation to inform targeted therapies.

The DDX5 (DEAD-box helicase 5) gene encodes a protein belonging to the DEAD-box RNA helicase family, which plays diverse and critical roles in multiple biological processes^29^. DDX5 is an ATP-dependent RNA helicase that plays a key role in RNA processing, transcriptional regulation, cell cycle control, and gene expression, and is implicated in the development of various diseases. It promotes RNA transcription, translation, and degradation by unwinding double-stranded RNA structures. Its multifaceted roles in tumourigenesis and tumour progression have been suggested^30^.

Among innovative candidates promoting pyroptosis and IL-18 secretion, amphibian derived host defence peptides, particularly caerin 1.1 and 1.9, have garnered attention for their multimodal antitumour activity. A 1:1 molar mixture of caerin 1.1 and 1.9 (caerin 1.1/1.9) has been shown to synergistically inhibit multiple tumour growth both in vitro and in vivo^31–33^. When coupled with radioactive isotope, the tumour inhibitory effect is significantly increased in multiple exogenic tumour models^34^. Moreover, combined with anti-PD1 and therapeutic vaccine, caerin 1.1/1.9 eradiates about 30% of tumour in both TC-1 and B16 tumour models^35^ mice completed eradicated the first tumour can resist 2nd tumour challenge^36^ and also inhibit distant tumour growth^32^. Mechanism studies demonstrated that caerin 1.1/1.9 activates tumour associated macrophages, promoting immunosuppressive M2 type macrophages reprogrammed to proinflammatory M1 type macrophages^37–39^. Furthermore, the antitumour responses mediated by caerin 1.1/1.9 is depended on the existence of macrophages^35^. ScRNA sequencing study unveiled that caerin 1.1/1.9 administration significantly changes the tumour microenvironment (TME)^38,39^ recruiting and activating immune cells, such as T cells, NK cells and professional antigen presenting cells to the tumour, specifically, recruiting cDC1 dendritic cells to the tumour, even to the distant tumour site^32^. Additionally, a recent study demonstrated that caerin 1.1/1.9 suppresses biosynthesis of both saturated and unsaturated fatty acids, while enhancing proinflammatory cytokine expression and inflammatory macrophage infiltration within the TME of a B16 melanoma mouse model^40^.

Recent mechanistic studies have revealed caerin 1.1/1.9 capacity to trigger pyroptosis in HPV positive cervical cancer cells. Notably, one-hour treatment with caerin 1.1/1.9 significantly upregulates gasdermin E (GSDME) expression in HeLa cells^41^ thereby enabling caspase-3-dependent pyroptosis characterized by plasma membrane ballooning, lactate dehydrogenase (LDH) release, and IL-18 secretion. This dual induction of apoptosis and pyroptosis positions caerin 1.1/1.9 as a multimodal therapeutic candidate against cancer. In this study, we aim to elucidate the role of KHDRBS1 in caerin 1.1/1.9-mediated pyroptosis in HeLa cells.

## Materials and methods

### Cells

The HeLa cell was obtained from the Shanghai Institute for Biological Sciences, Chinese Academy of Sciences. The GSDME KO HeLa cell line was provided by Dr Feng Shao (Chinese Academy of Biological Sciences, Beijing). The KHDRBS1 KO and DDX5 KO HeLa cell was purchased from the Yuanjing Biotech Inc., respectively.

### Peptide synthesis

Caerin 1.1 (referred to as F1, sequence: GLLSVLGSVAKHVLPHVVPVIAEHL-NH_2_), caerin 1.9 (referred to as F3, sequence: GLFGVLGSIAKHVLPHVVPVIAEKL-NH_2_), and a control peptide P3 without cytotoxic properties towards various cancer cells (GTELPSPPSVWFEAEFK-OH) were synthesised by Shanghai Qiang-yao Biological Technology Co., Ltd, China. The purity of the peptides was determined to be 95% via reverse-phase high-performance liquid chromatography. The caerin and P3 peptides were stored at 4°C until use. F1F3 was referred to the mixture of F1 and F3 at a molar ratio of 1:1.

### Cell viability assay

Cell viability is determined by MTT assay (Invitrogen) according to the manufacturer’s instructions. Briefly, 1.0 × 10^4^ of HeLa, KHDRBS1 KO HeLa, or DDX5 KO HeLa cells are cultured in 96-well plates overnight. The next day, different concentrations F1, F3, F1F3 (the mixture of F1 and F3 at a 1:1 molar ratio) were added and incubated at 37 °C with 5% CO_2_ overnight, followed by adding 10 µl of 5 mg/mL MTT reagent. After incubating for another 4 h, 50 µl of DMSO was added and the plates were measured at the OD540 nm using an enzyme-linked immunoassay instrument (Thermo Fisher).

### Quantitative PCR

HeLa, GSDME KO HeLa cells were cultured to F2 generation, 6-well plates were selected for seeding, and 1 × 10^6^/well was cultured for 18 h, F1F3 peptide stimulation for 1 h. In quantitative PCR experiments, total RNA is first extracted using Rapid Cell RNA Extraction Kit (GOONIE), followed by reverse transcription to generate cDNA (Takara). The synthesized cDNA serves as a template for PCR amplification, with SYBR Green nucleic acid probes used to monitor fluorescence intensity in each PCR cycle^42^. The relative expression levels of target genes are calculated by comparing the threshold cycle (Ct) values of fluorescence signals in different samples^43^.

### Enzyme-linked immunosorbent assay (ELISA)

HeLa, KHDRBS1 KO HeLa cells were cultured to F2 generation, 6-well plates were selected for seeding, and 1 × 10^6^/well was cultured for 18 h. The group was untreated, 10 µg/mL P3, F1, F3, F1F3 treatment group, and cell supernatants were collected after 1 h of stimulation. The levels of IL-1β and IL-18 in the cell supernatant were measured using a standard ELISA kit (Invitrogen) according to the manufacturer’s instructions.

### Lactate dehydrogenase (LDH) release assay

2 × 10^4^ cells of HeLa, GSDME KO HeLa were cultured in 96-well plates respectively and treated according to the manufacturer’s instructions. The culture supernatant was harvested and centrifuged at 600×*g* for 10 min. The supernatant of different groups was transferred to a new 96-well plate and assayed with the LDH Assay Kit following the manufactures’ instruction. The LDH activity is calculated as: (LDH sample − LDH low control)/(LDH high control - LDH low control) × 100%, where LDH sample, LDH low control, and LDH high control were OD450 measured with drug-treated, non-drug-treated, and lysate-treated supernatants (provided in the kit), respectively. Each sample is tested in triplicates.

### Sample preparation and cross-linking mass spectrometry (XL-MS)

HeLa cells (1 × 10^6^) were co-cultured with 10 µg/mL F1, F3, or P3 peptide PBS solutions at 37 °C for 45 min. The cells were homogenized, and the protein concentration was adjusted to 2.5 mg/mL. The homogenate was transferred into a crosslinking buffer (0.2 M triethanolamine, pH 8.0), followed by the addition of a 10-fold molar excess of the crosslinker dimethyl suberimidate (DMS). After incubation at room temperature for 45 min, the reaction was quenched by adding ice-cold acetic acid (1:4 ratio), and the samples were lyophilized for subsequent proteomic analysis.

The lyophilized samples were solubilized in 6 M urea buffer and subjected to ultrasonication. Reduction was performed using dithiothreitol (DTT, 37 °C, 60 min), followed by alkylation with iodoacetamide (room temperature, 60 min) and a second DTT quenching step (room temperature, 45 min). The urea concentration was then diluted, and trypsin (enzyme-to-substrate ratio 1:50) was added for overnight digestion at 37 °C. The reaction was terminated with 10% formic acid (pH < 3), and the resulting peptides were prepared for liquid chromatography-mass spectrometry (LC-MS/MS) analysis.

Tryptic peptides were analysed using an ExionLC system (AB SCIEX) coupled to a QTOF X500R mass spectrometer equipped with an electrospray ionization (ESI) source. Samples (20 µL) were separated on an Aeris PEPTIDE XB-C18 column (100 mm × 1.7 μm, Phenomenex) with a gradient elution: 5–35% solvent B (100% acetonitrile/0.1% formic acid, flow rate 400 µL/min) over 10 min, followed by a 2-min ramp to 80% B, a 1-min hold at 95% B for washing, and re-equilibration to initial conditions. MS parameters were set as follows: ion spray voltage 5500 V, declustering potential 100 V, curtain gas 30, ion source gases (GS1/GS2) 40/50, and temperature 450 °C. Data were acquired in information-dependent acquisition (IDA) mode, with TOF-MS scans (350–1400 m/z) triggering MS/MS (50–1800 m/z) for ions exceeding 100 cps intensity and charge states + 2 to + 5.

pLink2^44^ was used to analyse the cross-linking patterns of F1 and F3, with DMS as the linker molecule. The MS/MS data was searched against Homo sapiens (76,413 sequences, downloaded on Dec 12, 2014) database for protein identification, with the following search settings: enzyme trypsin; two missed cleavage sites; precursor mass tolerance 20 ppm; fragment mass tolerance 20 ppm; fixed modifications: Carbamidomethyl (C); variable modifications: oxidation (M), DMS (K), and deamidation (Q and N); and a cut-off of 1% FDR on peptide levels. The MS/MS spectra were visualized using pLabel.

### Overexpression of KHDRBS1 in HeLa cells

*Escherichia coli* harbouring the KHDRBS1 overexpression plasmid were cultured overnight in 10–15 mL LB medium under agitation (220 rpm, 37 °C). Plasmid DNA was subsequently extracted using a commercial purification kit (Invitrogen™ Plasmid Purification Kit) following the manufacturer’s instructions. For transfection, HeLa cells were plated in 12-well culture plates at a density of 2.4 × 10 cells per well and allowed to adhere overnight. Prior to transfection, the culture medium was replaced with serum- and antibiotic-free medium. Lipofe^5^ctamine™ 2000 transfection reagent (Invitrogen) was used to deliver plasmid DNA into cells at a mass-to-volume ratio of 1:2 (µg plasmid DNA: µL transfection reagent). The plasmid-transfection reagent complexes were incubated with cells for 5 h under standard culture conditions (37 °C, 5% CO_2_), after which the medium was replaced with complete medium supplemented with 10% fetal bovine serum and 1% penicillin-streptomycin. At 48 h post-transfection, cells were stimulated with 10 µg/mL F1F3 for 1 h. Finally, cell culture supernatants were harvested by centrifugation (2000 rpm, 5 min, 4 °C) and stored at − 80 °C for downstream analysis.

### Time-lapse imaging

To check the morphology of pyroptosis cells, 1 × 10^6^ cells of KHDRBS1 KO HeLa were cultured overnight in 24-well plates, respectively. After adding 10 µg/mL of F1F3 or P3, Olympus microscope (AP×100) was used for time-delay imaging immediately to observe the changes of cell morphology, cell membrane and cell nucleus.

### Mice

Female NSG mice (NOD.CB17-Prkdc^scid^ll2rg^tm1^/Bcgen), aged 6 to 8 weeks, were used in the experiment. These mice were purchased from the Zhuhai Bai Shi Tong Biotechnology Co., Ltd and housed under specific pathogen-free (SPF) conditions in the animal facility of the First Affiliated Hospital of Guangdong Pharmaceutical University. Each cage contained five mice, which were maintained in a controlled environment at 22 °C with 75% humidity and a 12-h light/dark cycle. They were provided with standard mouse chow and water ad libitum. All experiments were conducted in accordance with the guidelines provided by the Animal Experiment Ethics Committee (Ethical Approval No. GYFYG2R202326) and reported in compliance with ARRIVE (Animal Research: Reporting of In Vivo Experiments) guidelines.

### Tumour models

HeLa cells and KHDRBS1 KO HeLa cells (1 × 10^6^/200 µL) were injected subcutaneously into the flanks of NSG mice. Tumour volume is measured every two days using calipers and is calculated as follows: volume = length × (width^2^/2). When tumours are more than 15 mm in diameter, mice were judged as ethical death and humanely euthanised by carbon dioxide (CO_2_) inhalation.

### Tumour administration of caerin peptide

Three days after tumour cell inoculation (tumour diameter: 3–5 mm), mice were injected intratumourally with 30 µg of F1 or F3 peptide in PBS in 100 µL daily for 14 consecutive days.

### Hematoxylin and eosin (HE) staining

Tissue sections were dewaxed by immersion in xylene I and xylene II (15 min each), then rehydrated through a graded ethanol series: 100% ethanol I and II (5 min each), followed by 95%, 90%, 80%, 70%, and 50% ethanol (5 min each). Sections were then rinsed three times with distilled deionized water (ddH_2_O). Nuclear staining was performed using Harris hematoxylin for 20 min, after which slides were rinsed under tap water for 30 min to achieve bluing, with care taken to avoid direct water stream impact on tissue sections. Following three ddH_2_O washes, sections were dehydrated sequentially in 50%, 70%, 80%, and 90% ethanol (2 min each). Cytoplasmic counterstaining was conducted with eosin Y solution for 10 s, followed by final dehydration in 95% ethanol I and II (2 min each), then 95% ethanol II (2 min), 100% ethanol I (5 min), 100% ethanol II (5 min) and clearing in xylene I and xylene II (5 min each). Slides were permanently mounted with neutral resin and examined under a ZEISS AX10 light microscope for image acquisition.

### Detection of cell surface makers by flow cytometry

To obtain single cells, HeLa, KHDRBS1 KO HeLa tumour tissues were isolated and homogenised with a tumour dissociation kit (Miltenyi Biotec, Bergisch Gladbach, Germany). Subsequently, single cells were stained with different antibodies, as described below. Viable cells were analysed using a flow cytometer (FACS Aria II; BD Biosciences, San Jose, CA, USA). Data analysis was performed using Flow Jo v10.0 software (Tree Star Inc., Ashland, OR, USA).

### Flow cytometry

Antibodies used for flow cytometry are listed in Table 1.

**Table 1:** Flow cytometry antibodies.

Antibody	Label	Article number	Clone	Provider
CD45.2	FITC	11-0454-85	104	eBioscience
F4/80	PE	12-4801-82	BM8	eBioscience
CD11b	Percp-cy5.5	45-0112-82	M1/70	eBioscience
CD8a	PE-cy7	552,877	53 − 6.7	BD Bioscience
CD3e	APC-cy7	557,596	145-2C11	BD Bioscience
Ly6C	BV421	562,727	AL-21	BD Bioscience
Fixable viability stain 510		564,406		BD Bioscience
Ly-6G	APC	17-9668-82	1A8-Ly6g	eBioscience

### Multiplex immunofluorescence sample pretreatment and staining

Tissue sections of 3 μm thickness were cut from paraffin-embedded tissue blocks and transferred to a 40 °C water bath for floating. After complete flattening, sections were mounted onto glass slides. The slide-mounted sections were then dried overnight at 42 °C for section spreading or alternatively baked at 65 °C for 1 h. For dewaxing, slides were vertically positioned in xylene and incubated for 3 h. Subsequent rehydration was performed through sequential immersion in absolute ethanol, 95% ethanol, 70% ethanol, 50% ethanol, and pure water (20 min each). Antigen retrieval was conducted by incubating slides in alkaline antigen retrieval buffer at 95 °C for 20 min, followed by 40 min of natural cooling to room temperature. The retrieved slides were then stored in PBS buffer at 4 °C. For autofluorescence quenching, slides were immersed in 3% hydrogen peroxide solution and exposed to 50 W LED light for two 45-min intervals (with solution replacement between exposures). Following two PBS washes, slides were maintained in PBS buffer. Permeabilization was achieved by incubating slides in PBS containing 0.5% Triton X-100 for 15 min at room temperature, followed by two PBS washes before storage in PBS buffer. Finally, slides were processed using the MGISEQ-2000RS FluoXpert system with the corresponding FluoXpert multiplex immunofluorescence reagent kit. Stained images were acquired and visualized through the FluoXpert staining software interface.

### Statistical analysis

The flow experimental data was analysed using Flowjo 10 software. The results were expressed as the mean ± Standard error, and the differences between groups were analysed using GraphPad Prism 9 software. Calculate statistical differences between groups using t-tests, or one-way ANOVA.

## Result

### F1F3-induced pyroptosis in HeLa cells is independent of GSDME

**Fig. 1:**
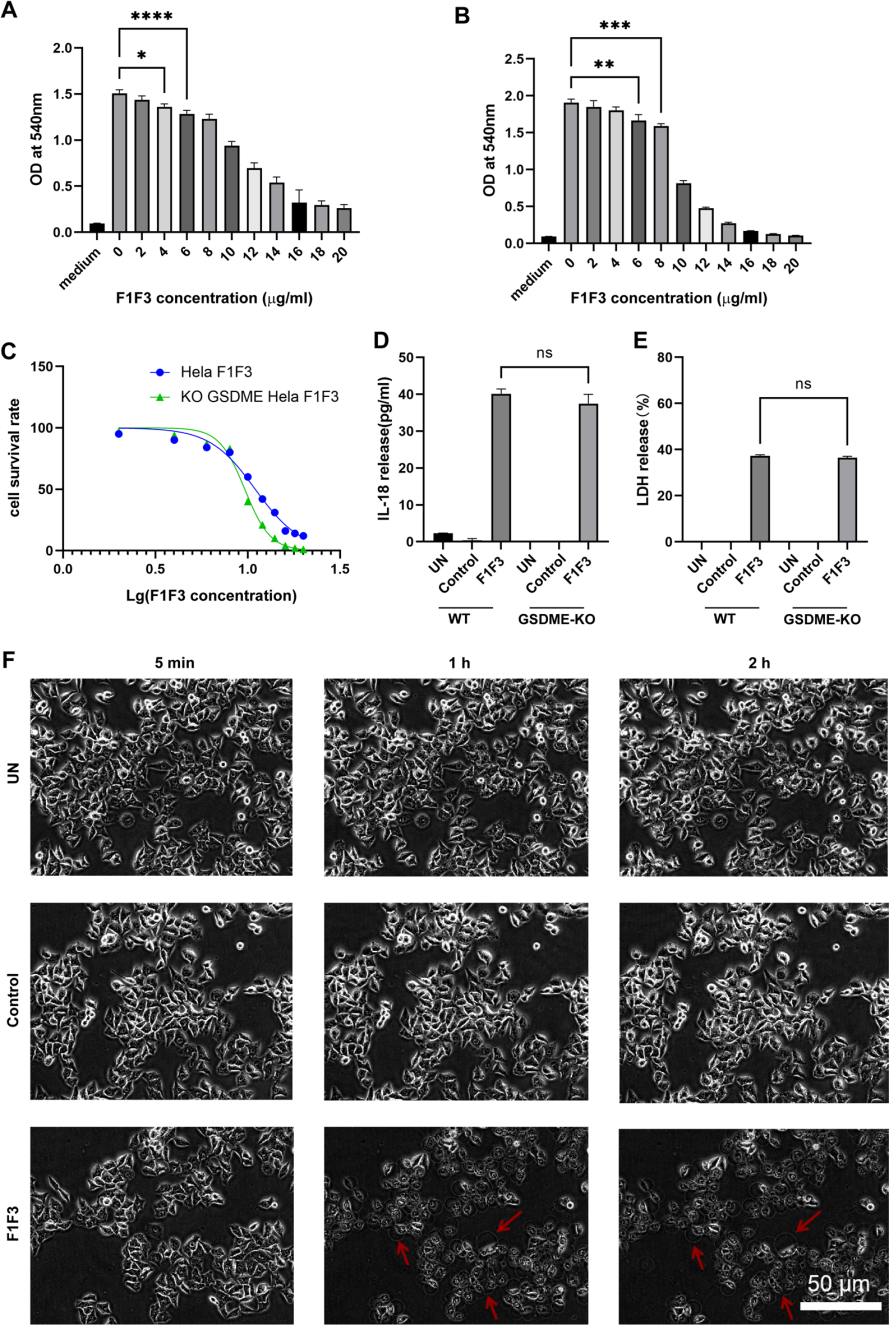
F1F3 peptide suppresses proliferation and induces cytotoxicity in wild-type and GSDME-knockout HeLa cells. Cytotoxic assays showing does-dependent decreases in cell viability following F1F3 treatment in wild-type (WT) HeLa cells (**A**) and GSDME-knockout (KO) HeLa cells (**B**). X-axis: F1F3 concentration; Y-axis: OD at 540 nm. (**C**) IC_50_ values of F1F3 peptide in WT and GSDME-KO HeLa cells were 10.98 µg/mL and 9.64 µg/mL, respectively. (**D**) IL-18 secretion levels measured 1 h post-F1F3 stimulation in WT and GSDME-KO cells. (**E**) LDH release assays indicating membrane damage after 1 h of F1F3 treatment in both cell types. (**F**) Real-time fluorescence imaging of GSDME-KO HeLa cells within 2 h post-treatment to visualize dynamic responses. Data are presented as mean ± standard error of the mean (SEM). Statistical analysis was performed using one-way analysis of variance (ANOVA): ns (not significant), *P < 0.05, **P < 0.01, ***P < 0.001, ****P < 0.0001.

Previously, we demonstrated that the treatment of HeLa cells with F1F3 for 1 h induced features characteristic of pyroptosis, including cell membrane swelling, bubble formation, LDH release and IL-18 secretion. Furthermore, IL-18 secretion was dose dependent and significantly higher in HeLa cells treated with F1F3 compared to cells treated with either F1 or F3 along. Upregulation of GSDME and cleaved caspase 3 expression was also observed, suggesting that F1F3 may induce pyroptosis via the caspase 3/GSDME axis^45^. To verify this hypothesis, GSDME knockout (KO) HeLa cells were generated, and the absence of GSDME expression was confirmed by Western blot (Fig. S1A). MTT assays showed that F1F3 significantly inhibited the proliferation of both HeLa cells and GSDME KO HeLa cells. The half maximal inhibitory concentration (IC50) of F1F3 for wild-type HeLa cells was 10.98 µg/mL, while the IC50 for GSDME KO HeLa cells was 9.64 µg/mL (Fig. 1A–C). The similar IC50 values indicated comparable cytotoxicity between the two cell types. Further analysis of IL-18 and LDH release in HeLa cells and GSDME KO HeLa cells showed no significant difference between wild-type and GSDME KO HeLa cells following F1F3 treatment (Fig. 1D,E). To investigate the morphological changes associated with pyroptosis, GSDME KO HeLa cells were treated with 10 µg/mL of either a control peptide (P3), F1F3, or left untreated, and observed in real time for 2 h using live-cell imaging (Fig. 1F). While untreated and P3-treated GSDME KO cells maintained normal morphology, F1F3-treated GSDME KO cells exhibited clear signs of pyroptosis, including cell membrane swelling and bubble formation. Together, these results suggest that the F1F3 induces pyroptosis in HeLa cells through a GSDME-independent mechanism. To further investigate the underlying mechanisms of F1F3 induced pyroptosis in GSDME KO HeLa cells, the expression profiles of gasdermin family genes were examined by qPCR following 1 h F1F3 stimulation. The qPCR results demonstrated no significant alterations in the transcriptional levels of GSDMA, GSDMB, GSDMC, GSDMD, or PJVK (Fig. S1B–F). The primer sequences used in the qPCR experiments are shown in Table 2.

**Table 2:** Primer sequences.

Name	Sequence
qh-GSDMA-F1	CACCTAAGGGCATTTCAGA
qh-GSDMA-R1	TCAGCAGATGGGCTTAGTG
qh-GSDMB-F1	AGCTGAGGTCAGAGAGGAGT
qh-GSDMB-R1	TCCCTTTGGCCCTTGTGTTT
qh-GSDMC-F1	GTGGTGCCATCCTAAAGAAA
qh-GSDMC-R1	GCAGGATCCTCTTCTCCAT
qh-GSDMD-F1	AGCCAGAACACAAAGTCCTG
qh-GSDMD-R1	AGAAGGACGTCCAAGTCAGA
qh-PJVK-F1	CCCTAGGCCGCAGTTCTTTG
qh-PJVK-R1	ACTTTAGGCCTCAACGACCC

### XL-MS indicated F1 binds KHDRBS1, while F3 binds DDX5

**Fig. 2:**
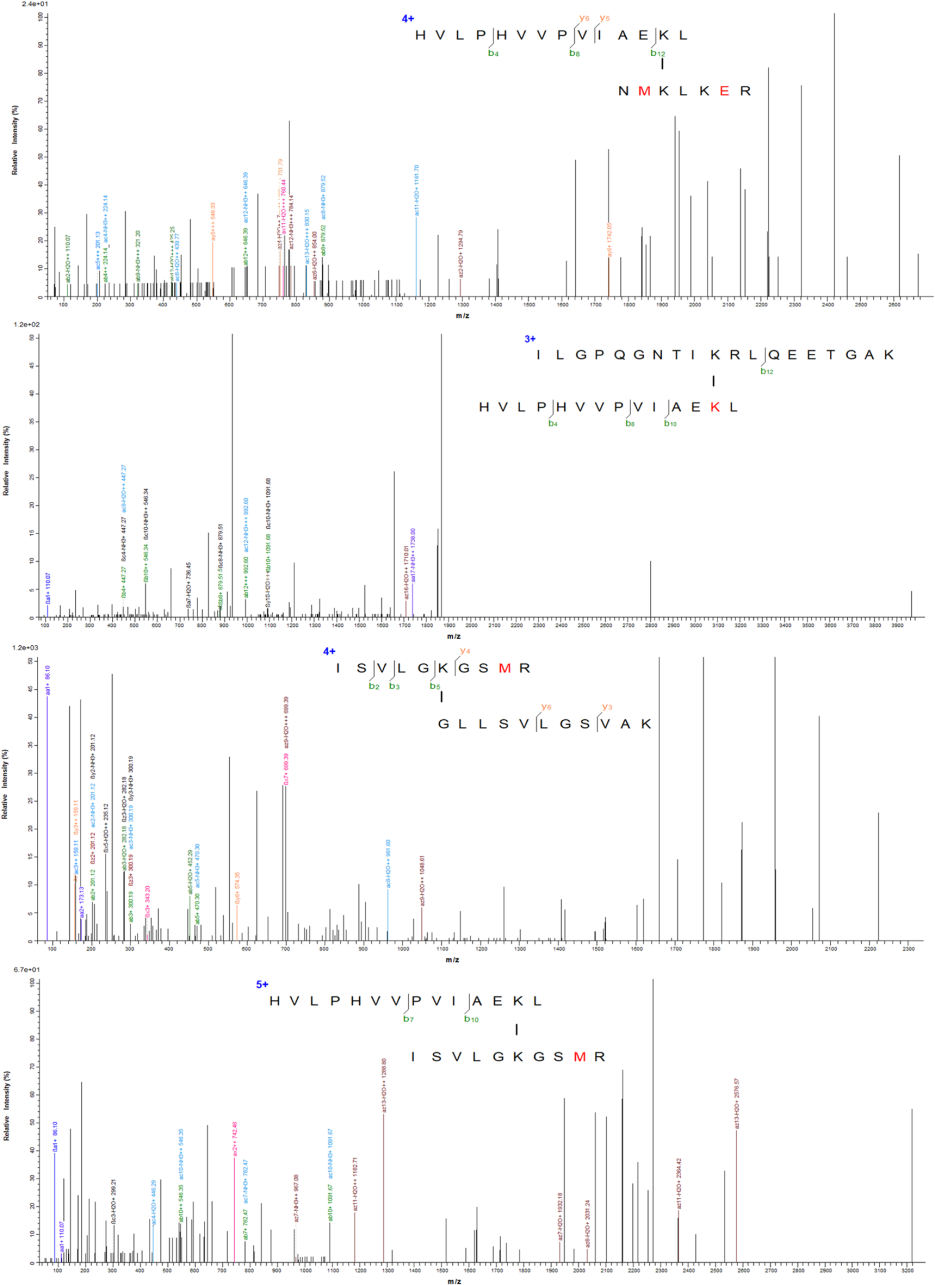
MS/MS spectra showing F1–KHDRBS1 interactions identified by cross-linking mass spectrometry. Cross-linking mass spectrometry (XL-MS) was performed using DMS as the crosslinker to identify direct interaction sites between the F1 peptide and KHDRBS1 protein in HeLa cells. MS/MS spectra revealed specific crosslinks between F1 and three lysine residues on KHDRBS1, including Lys155, Lys185, and Lys200. These findings suggest multiple contact points and support a direct interaction between F1 and KHDRBS1.

F1F3-treated HeLa cells exhibited pyroptosis even in the absence of GSDME, and no compensatory upregulation of other gasdermin family members was detected. Notably, IL-18 secretion levels were comparable between wild-type and GSDME-knockout HeLa cells following F1F3 treatment. These findings suggest that F1 and F3 may act through GSDME-independent pathways, prompting further investigation into their cellular targets. To identify potential binding partners, we employed crosslinking mass spectrometry (XL-MS) in HeLa cells. The results revealed that F1 crosslinked with KHDRBS1 at multiple lysine residues, including Lys155, Lys185, and Lys200 (Fig. 2), indicating potential interaction sites^46^. F3 was found to interact with DDX5 at Lys490 (Fig. S2). In contrast, a control peptide (P3) did not show binding to any proteins under the same conditions.

### KHDRBS1 knockout (KO) HeLa cells and DDX5 KO HeLa cells are more sensitive to F1F3

**Fig. 3:**
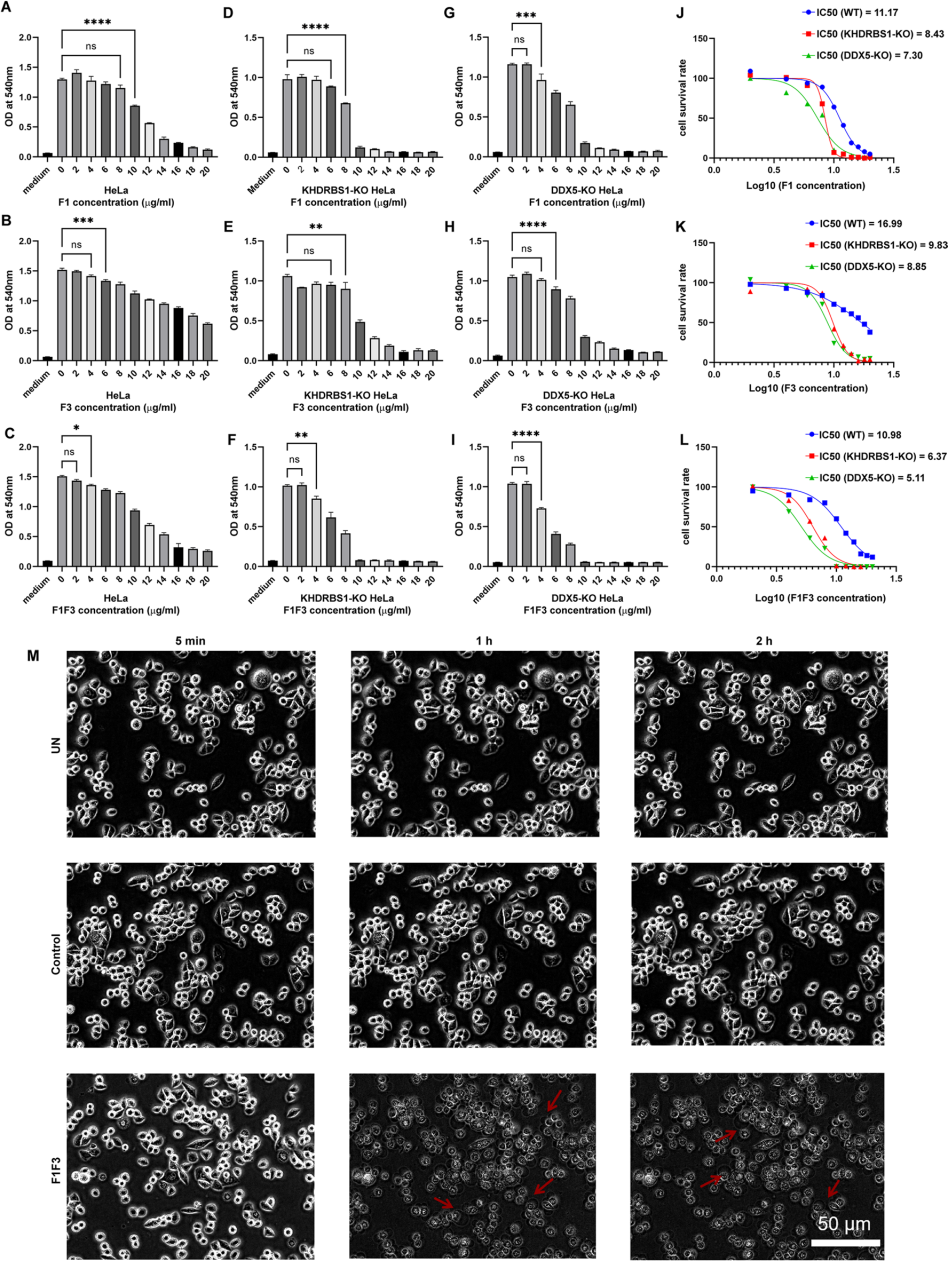
KHDRBS1- and DDX5-knockout HeLa cells exhibit increased sensitivity to F1F3 treatment. (**A-C**) Dose-dependent cytotoxicity of F1, F3, and F1F3 polypeptides in wild-type HeLa cells, as measured by optical density (OD_540_ nm). (**D-F**) Cytotoxicity assays in KHDRBS1-KO HeLa cells treated with F1, F3, or F1F3 showed enhanced sensitivity, indicated by a greater reduction in OD_540_ nm. (**G-I**) Similar dose-dependent cytotoxicity was observed in DDX5-KO HeLa cells. (**J-L**) Calculated IC₅₀ values for F1, F3, and F1F3 across wild-type, KHDRBS1-KO, and DDX5-KO HeLa cells demonstrated increased potency of the peptides in KO cell lines. (M) Real-time fluorescence imaging of KHDRBS1-KO HeLa cells within 2 h post-F1F3 treatment revealed dynamic cellular responses. (**A-L**) Data are presented as mean ± SEM. Statistical significance was evaluated using one-way ANOVA: ns (not significant), *P < 0.05, **P < 0.01, ***P < 0.001, ****P < 0.0001.

Next, KHDRBS1 and DDX5 in HeLa cells were knockout using CRISPR-Cas9 technology. Wild type HeLa cells, KHDRBS1 KO HeLa cells, and DDX5 KO HeLa cells were treated with F1, F3 or F1F3 respectively for 18 h and MTT assay was employed to examine whether these cells responded differently to F peptides treatment. MTT assays showed that F1, F3, and F1F3 significantly inhibited the proliferation of HeLa, KHDRBS1 KO HeLa, and DDX5 KO HeLa cells. The half-maximal inhibitory concentrations (IC_50_) of F1, F3, and F1F3 for HeLa cells were 11.17 µg/mL, 16.99 µg/mL, and 10.98 µg/mL, respectively (Fig. 3A–C). The IC_50_ values of F1, F3, and F1F3 for KHDRBS1 KO HeLa cells were 8.43 µg/mL, 9.83 µg/mL, and 6.37 µg/mL, respectively (Fig. 3D–F). The IC_50_ values of F1, F3, and F1F3 for DDX5 KO HeLa cells were 7.30 µg/mL, 8.85 µg/mL, and 5.11 µg/mL, respectively (Fig. 3G,I). The combined use of F1 and F3 showed a better inhibitory effect. KHDRBS1 KO HeLa and DDX5 KO HeLa cells were more sensitive to the F1F3 peptide than HeLa cells (Fig. 3J–L). We assessed the morphological effects of F1F3 peptide stimulation on KHDRBS1 KO HeLa cells. Cells were treated with untreated medium, 10 µg/mL of P3, or 10 µg/mL of F1F3, and observed continuously for 2 h using an Olympus microscope (AP×100) to evaluate cellular morphological changes (Fig. 3M). The results showed that the cell morphology and cell membrane of untreated and P3-treated KHDRBS1 KO HeLa cells remained unchanged. In contrast, F1F3-treated KHDRBS1 KO HeLa cells exhibited significant cell membrane swelling and bubbles after treatment. Cell swelling and the production of numerous bubbles are additional morphological features of pyroptosis, further confirming that the peptide promotes pyroptosis in KHDRBS1 KO HeLa cells.

### KHDRBS1 deficiency enhances IL-18 secretion in HeLa cells following F1F3 treatment

**Fig. 4:**
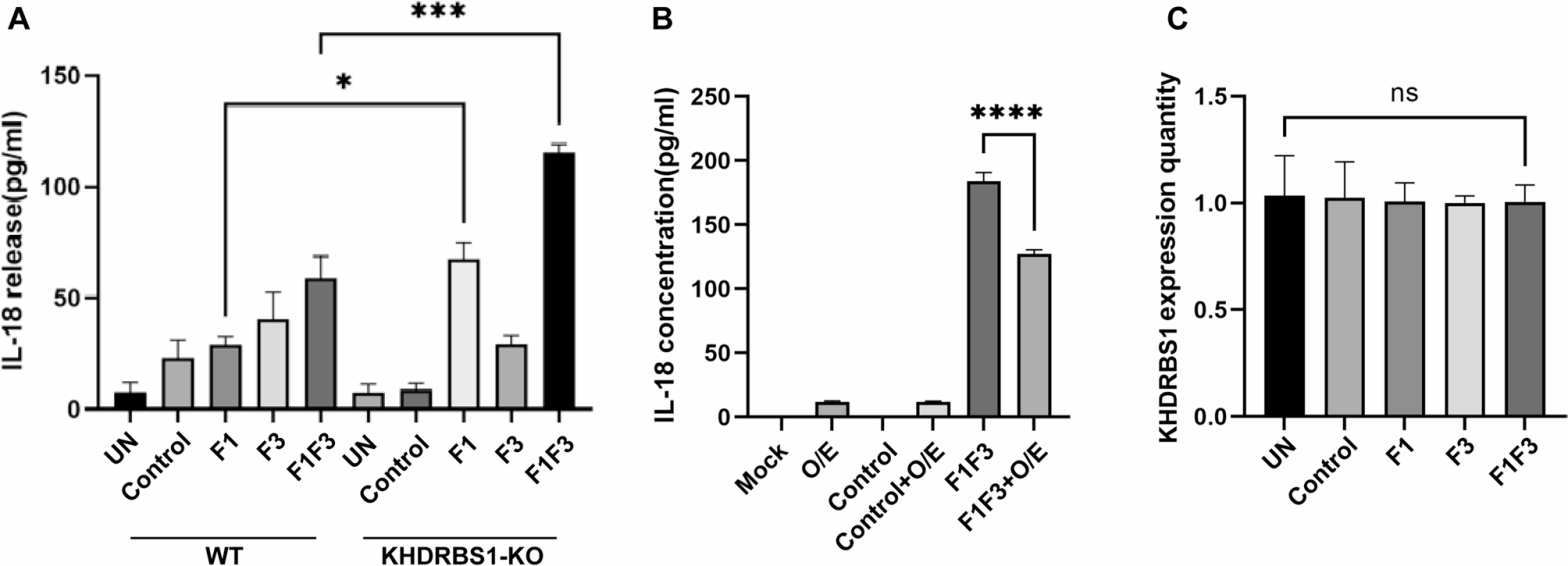
The presence of KHDRBS1 suppresses IL-18 secretion under F1F3 stimulation. (**A**) IL-18 release levels were quantified in wild type HeLa cells and KHDRBS1 KO HeLa cells following 1-hour stimulation with P3, F1, F3, or the F1F3 polypeptide combination. (**B**) To further elucidate the regulatory role of KHDRBS1, plasmid-mediated overexpression of KHDRBS1 was performed in HeLa cells. IL-18 secretion was subsequently measured by ELISA after 1-hour treatment with P3 or F1F3. (**C**) The expression of KHDRBS1 in HeLa cells after 1-hour stimulation with F1F3 was analyzed by qPCR. Data are presented as mean ± SEM. Statistical significance was evaluated using one-way ANOVA, with *P < 0.05, ***P < 0.001, and ****P < 0.0001.

HeLa and KHDRBS1 KO HeLa cells were treated with 10 µg/mL of F1F3 peptide for 1 h, and the supernatants were collected to measure IL-18 secretion by ELISA. The results showed that KHDRBS1 KO HeLa cells released more IL-18 compared to wild type HeLa cells (Fig. 4A), suggesting that KHDRBS1 negatively regulates IL-18 secretion in response to F1F3 stimulation. To further confirm this observation, a KHDRBS1-expressing plasmid was transfected in HeLa cells, followed by treatment with 10 µg/mL of F1F3 for 1 h. IL-18 levels in the culture supernatant were measured by ELISA. Successful transfection was confirmed by live-cell imaging using a laser scanning confocal microscope (e.g., Zeiss LSM 880) equipped with environmental controls (Fig. S3A). Cells were maintained in PBS during imaging to preserve viability. Transfection efficiency was calculated by dividing the number of fluorescent cells by the total number of cells in the blank control group (Fig. S3B). The results showed that under F1F3 stimulation, KHDRBS1-overexpressing HeLa cells secreted significantly less IL-18 compared to non-transfected cells (Fig. 4B), further supporting that KHDRBS1 suppresses IL-18 release upon F1F3 treatment. To determine whether F1F3 stimulation affects KHDRBS1 expression itself, we performed qPCR using specific primers following a 1 h treatment with F1, F3, or the F1F3 combination. The analysis revealed no significant changes in KHDRBS1 mRNA expression across treatment groups compared to the untreated control (Fig. 4C).

### KHDRBS1 KO accelerates tumour growth and increases sensitivity to F1F3 in NSG mice

**Fig. 5:**
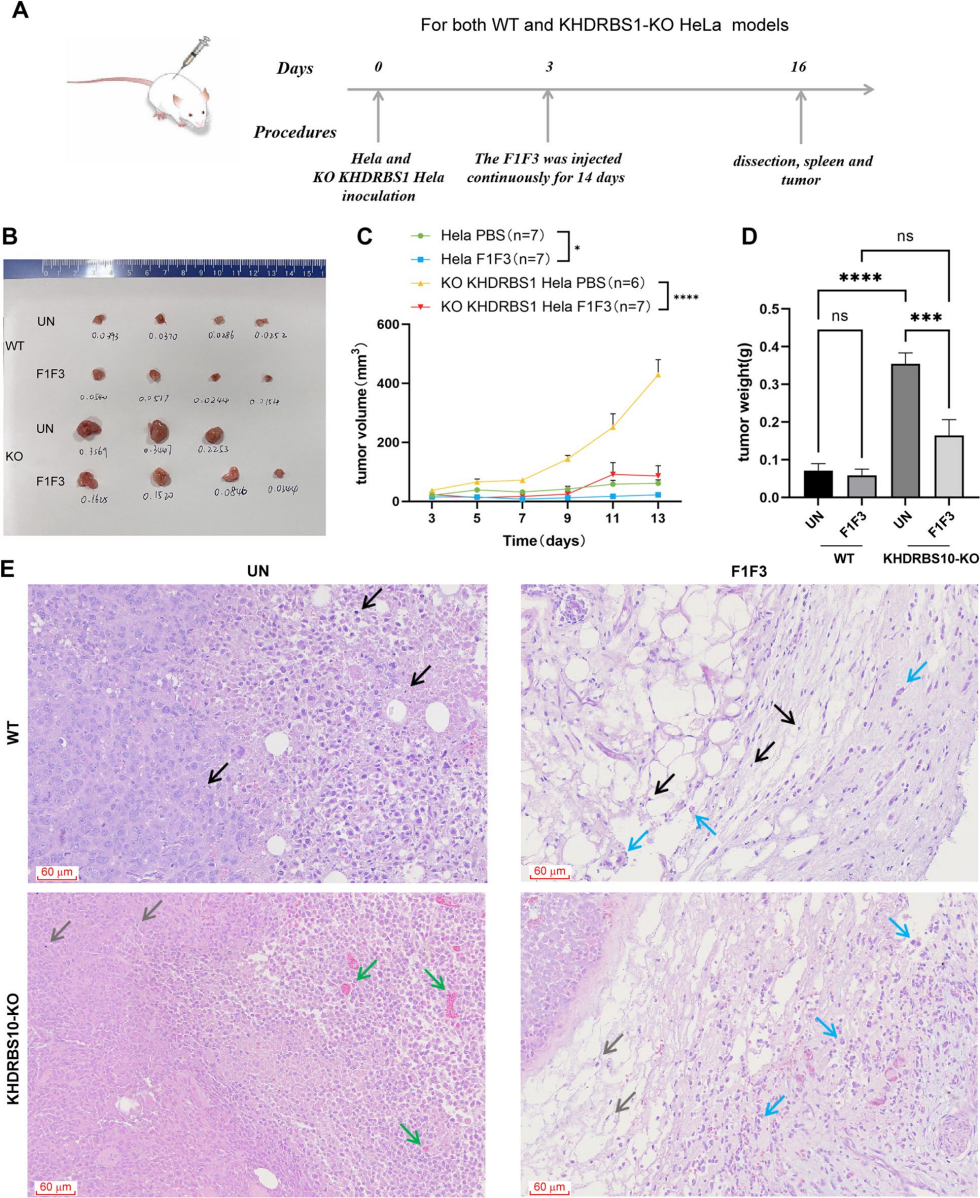
KHDRBS1 mediates suppression of F1F3-induced IL-18 secretion in vivo. (**A**) Schematic of the NSG mouse therapeutic model used for evaluating F1F3 treatment efficacy. (**B**–**C**) Tumour weight and size measurements of xenografts from treated and control groups. (**D**) Comparison of tumour weights derived from wild-type HeLa cells and KHDRBS1-KO HeLa cells. (**E**) Hematoxylin and eosin (H&E) staining of tumour tissues illustrating histopathological differences between groups. (**B**–**E**) Data are presented as mean ± SEM. Statistical significance was assessed by one-way ANOVA: ns (not significant), *P < 0.05, ****P < 0.0001.

To further investigate the effects of F1F3 on tumour growth in vivo, a xenograft model was established in NSG mice by subcutaneously transplanting either wild-type HeLa cells or KHDRBS1 KO HeLa cells into the flanks of the mice. Once tumours were established, intratumoral injections of F1F3 were administered (Fig. 5A). On Day 16, two days after the final treatment, tumours were excised and weighted. The results showed that, compared with the PBS treated group, F1F3 significantly inhibited the growth of KHDRBS1 KO HeLa tumours, but not wild-type HeLa tumours (Fig. 5B,D). There was no significant difference in tumour weight between the F1F3 and the PBS groups in the wild-type HeLa tumour model (Fig. 5C), consistent with our previous findings in nude mice^34^ suggesting that F1F3 requires components of an intact immune system to exert anti-tumour effects. In contrast, KHDRBS1 KO HeLa tumours in the F1F3 group exhibited significantly reduced tumour weights compared to the PBS group (Fig. 5D), indicating increased sensitivity of KHDRBS1-deficient tumours to F1F3 treatment, which was consistent with our in vitro findings (Fig. 3C,F,L). Moreover, comparison of tumour sizes in the PBS group showed that KHDRBS1 KO tumours were larger than wild-type HeLa tumours.

Histopathological analysis revealed notable morphological differences between groups. In the HeLa PBS group, patchy tumour necrosis was observed without significant inflammatory cell infiltration (black arrows). In contrast, the HeLa F1F3 group exhibited extensive tumour cell necrosis, accompanied by mild infiltration of macrophages within the surrounding connective tissue (blue arrows). The KHDRBS1 KO HeLa PBS group displayed mitotic figures (grey arrows) and vascular congestion (green arrows), with no notable abnormalities in inflammatory cell infiltration. However, in the F1F3-treated KHDRBS1 KO group, tumour tissue displayed active mitoses (grey arrows) along with increased macrophage presence (blue arrows) in the peritumoral connective tissue (Fig. 5E). These findings indicate that stimulation with the F1F3 significantly enhances macrophage infiltration, potentially contributing to modulation of the TME, particularly in the absence of KHDRBS1.

**Fig. 6:**
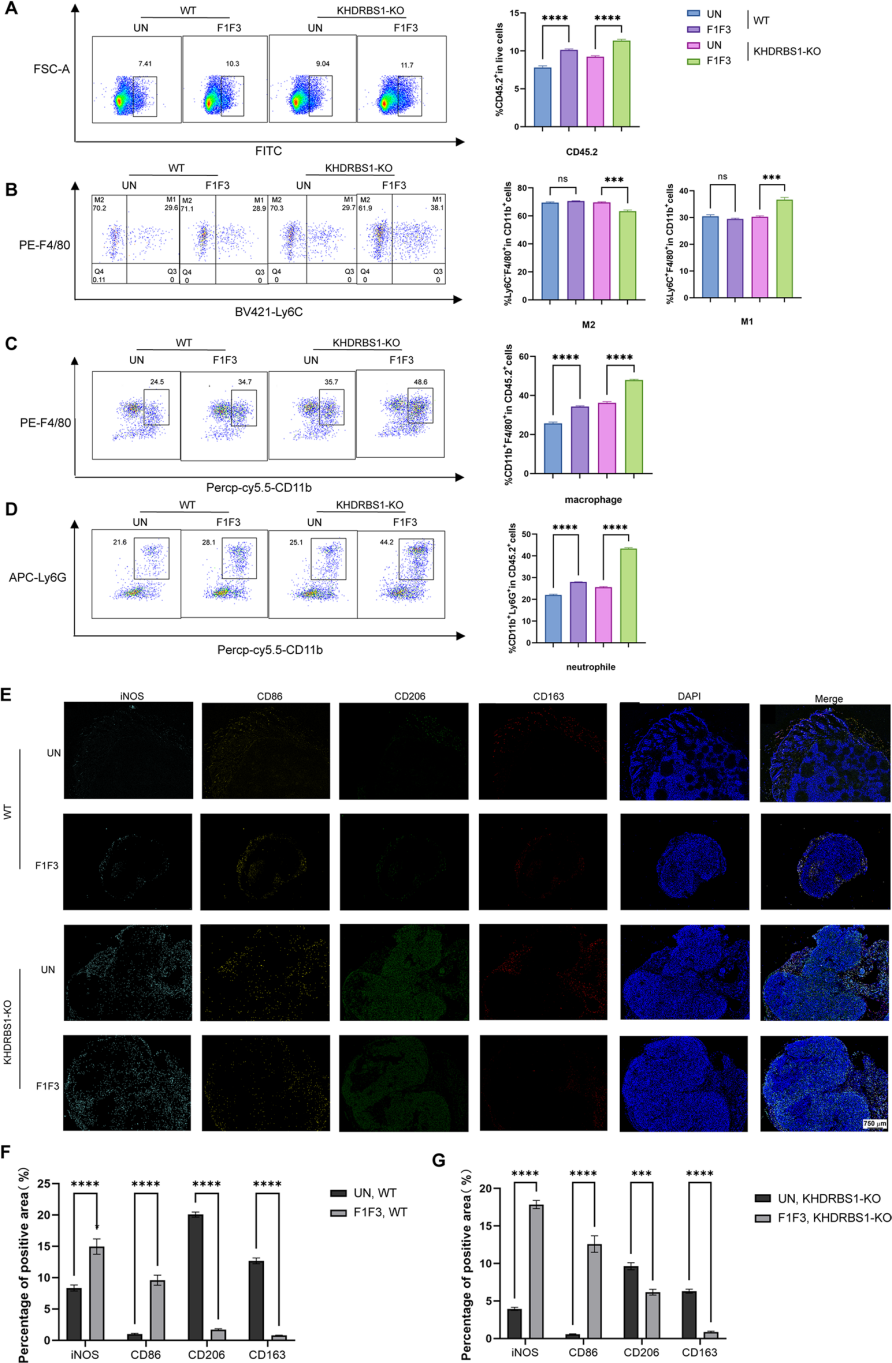
F1F3 modulates the splenic immune microenvironment in tumour-bearing mice with HeLa and KHDRBS1-knockout HeLa xenografts. Flow cytometry was used to evaluate immune cell populations in the spleens of different treatment groups: (**A**) Total immune cells (CD45.2^+^), (**B**) M1 macrophages (CD45.2^+^CD11b^+^Ly6C^+^) and M2 macrophages (CD45.2^+^CD11b^+^Ly6C^−^), (**C**) Total macrophages (CD45.2^+^CD11b^+^F4/80^+^), (**D**) Neutrophils (CD45.2^+^PerCP-Cy5.5^+^Ly6G^+^). (**E**) Representative multiplex immunofluorescence images of tumour tissues. (**F**) F1F3-induced changes in M1 and M2 macrophage markers in wild-type HeLa tumours. (**G**) Corresponding changes in M1 and M2 markers in KHDRBS1-KO HeLa tumours following F1F3 treatment. (**A**–**D**, **F**–**G**) Data are shown as mean ± SEM. Statistical significance was assessed by one-way ANOVA: ns (not significant), ***P < 0.001, ****P < 0.0001.

Subsequently, immune cell infiltration profiles were analyzed by flow cytometry. Notably, intratumoral injection of F1F3 significantly increased the proportions of CD45.2^+^ leukocytes, macrophages, and neutrophils in spleens derived from both HeLa and KHRBS1 KO HeLa tumour models compared with PBS-treated controls (Fig. 6A–D). This treatment concurrently elevated M1-like macrophage polarization while reducing M2-like macrophage populations, consistent with previous observations in B16 and TC-1 tumour models^35,39^.

Furthermore, multiplex immunofluorescence staining was employed to simultaneously visualise multiple biomarkers in situ within tissue sections (Fig. 6E). Following F1F3 stimulation, both HeLa and KHDRBS1 KO HeLa tumours showed strong upregulation of iNOS (inducible nitric oxide synthase) and CD86, canonical markers of M1 macrophages with pro-inflammatory and antitumour (Fig. 6F,G). In contrast, expression of CD206 and CD163, markers associated with M2 macrophages and their immunosuppressive, pro-tumourigenic roles, was markedly downregulated. These findings indicate that F1F3 stimulation promotes macrophage polarization toward the M1 phenotype while suppressing the M2 population in the TME, thus suggesting the immunomodulatory and therapeutic potential of F1F3 in tumour immunity.

## Discussion

In this study, we demonstrated that F1F3 mediated pyroptosis-like cell death in HeLa cells operates independently of the canonical GSDME pathway, although our previous findings indicated that F1F3 treatment upregulates cleaved caspase-3 and GSDME expression. Investigation using GSDME knockout (KO) HeLa cells revealed that key pyroptotic features, including cell membrane blubbing, release of LDH, and secretion of IL-18, remain unchanged in GSDME KO HeLa cells (Fig. 1). Moreover, quantitative PCR analysis showed no compensatory upregulation of other gasdermin family members, suggesting that F1F3-induced cell death represents a noncanonical, GSDM-independent pyroptosis-like phenotype that warrants deeper mechanistic exploration.

XL-MS discovered that F1 binds to KHDRBS1, while F3 binds to DDX5 (Fig. 2). Further experiments in KHDRBS1 or DDX5 KO HeLa cells unveiled that both KHDRBS1 KO and DDX5 KO HeLa cells are more sensitive to F1F3 (Fig. 3); Interestingly, secretion of IL-18 following F1F3 treatment was increased in KHDRBS1 KO HeLa cells (Fig. 4). Moreover, KHDRBS1 KO HeLa cells grow faster in NSG mice compared with wild type HeLa cells and respond to F1F3 in NSG mice that lack NK cells and adaptive immune cells (Fig. 5).

Pyroptosis, a form of pro-inflammatory programmed cell death, is mechanistically characterized by the activation of GSDM proteins, which induces plasma membrane rupture and subsequent release of pro-inflammatory mediators, thereby eliciting robust inflammatory cascades^47^. Accumulating evidence demonstrates that pyroptosis orchestrates immune system activation through the liberation of damage-associated molecular patterns (DAMPs) and pro-inflammatory cytokines, playing pivotal roles in antimicrobial defence, antitumour immunity^48,49^. Notably, pyroptotic processes have been shown to promote dendritic cell activation and antigen presentation efficiency, thereby potentiating adaptive immune responses^50^. Furthermore, emerging studies highlight the critical involvement of pyroptosis in TME remodelling, where the release of inflammatory cytokines and immunomodulatory molecules exerts dual effects on tumour suppression and enhancement of immunotherapeutic efficacy^51^. However, our findings challenge the conventional understanding of pyroptosis in cancer immunotherapy by demonstrating a GSDME-independent mechanism of tumour cell death with retained immunostimulatory features, including IL-18 secretion, although further investigation is needed to confirm current findings.

IL-18, a critical immunomodulatory cytokine, exhibits pleiotropic and context-dependent functions in tumour immunology. IL-18 exerts tumour-suppressive effects through Th1-type immune response activation and augmentation of cytotoxic T lymphocyte functionality, thereby promoting antitumour immunity^52^. Mechanistically, IL-18 stimulates IFN-γ production, which subsequently activates natural killer cells and CD8^+^ T lymphocytes, significantly enhancing their tumour-specific cytolytic activity^53^. Moreover, IL-18 demonstrates pro-inflammatory properties in select malignancies, suppressing tumour progression through angiogenesis inhibition and facilitation of immune cell infiltration within the TME^54^. Emerging evidence, however, reveals the functional dichotomy of IL-18 across heterogeneous cancer types, with its biological outcomes being contingent upon tumour-specific genomic landscapes and microenvironmental cues. In pancreatic ductal adenocarcinoma, elevated IL-18 expression correlates with unfavourable prognosis, potentially mediated through tumour cell invasiveness potentiation and immune evasion mechanisms^55^.

Conversely, in malignancies such as renal cell carcinoma and breast cancer, IL-18 manifests prototypical antitumour characteristics, including enhanced immune surveillance and tumour cell apoptosis induction^56^. This functional duality underscores IL-18’s capacity to either promote tumour eradication through immune effector cell activation or paradoxically facilitate tumour progression via microenvironmental reprogramming, necessitating precision-based evaluation of its therapeutic modulation^20^. In this study, we identified KHDRBS1 as a novel regulator of IL-18 secretion. KHDRBS1 knockout significantly enhanced IL-18 release following F1F3 treatment, and ectopic overexpression of KHDRBS1 in wild-type HeLa cells suppressed this secretion (Fig. 4A,B). These results, to our knowledge, are the first to link KHDRBS1 to the modulation of IL-18 production.

Using cross-linking mass spectrometry, we discovered that F1 directly binds KHDRBS1, while F3 targets DDX5 (Fig. 2). The interaction between F1 and KHDRBS1 disrupts its cytoplasmic associations with key signalling proteins, such as Src, Lck, Grb2, and Bcl-xL^57–59^ thereby impairing its role in survival and proliferation pathways. Additionally, this interaction interferes with KHDRBS1’s nuclear translocation and its function as a transcriptional repressor of Wnt signalling^60^ collectively resulting in suppressed cell proliferation and the induction of cell death.Functional validation showed that both KHDRBS1 KO and DDX5 KO HeLa cells exhibited heightened sensitivity to F1F3-induced cytotoxicity (Fig. 3C,F,I,L). KHDRBS1 and DDX5 are multifunctional RNA-binding proteins involved in transcriptional regulation, splicing, and cell survival signalling. Loss of KHDRBS1 may dysregulate splicing of pro-survival genes and disrupt MAPK and PI3K/AKT pathways, thereby sensitizing cells to F1F3. Similarly, DDX5 depletion may impair ribosome biogenesis and transcriptional coactivation of oncogenic targets (e.g., MYC, BCL-2), compounding cellular stress upon F1F3 challenge. These findings position KHDRBS1 and DDX5 as modulators of F1F3 efficacy and potential biomarkers of therapeutic responsiveness. Currently, we are investigating whether KHDRBS1 inhibits IL-18 secretion by other stimulators, such as LPS and TNFα, which have been demonstrated to increase the IL-18 secretion^61,62^.

**Fig. 7:**
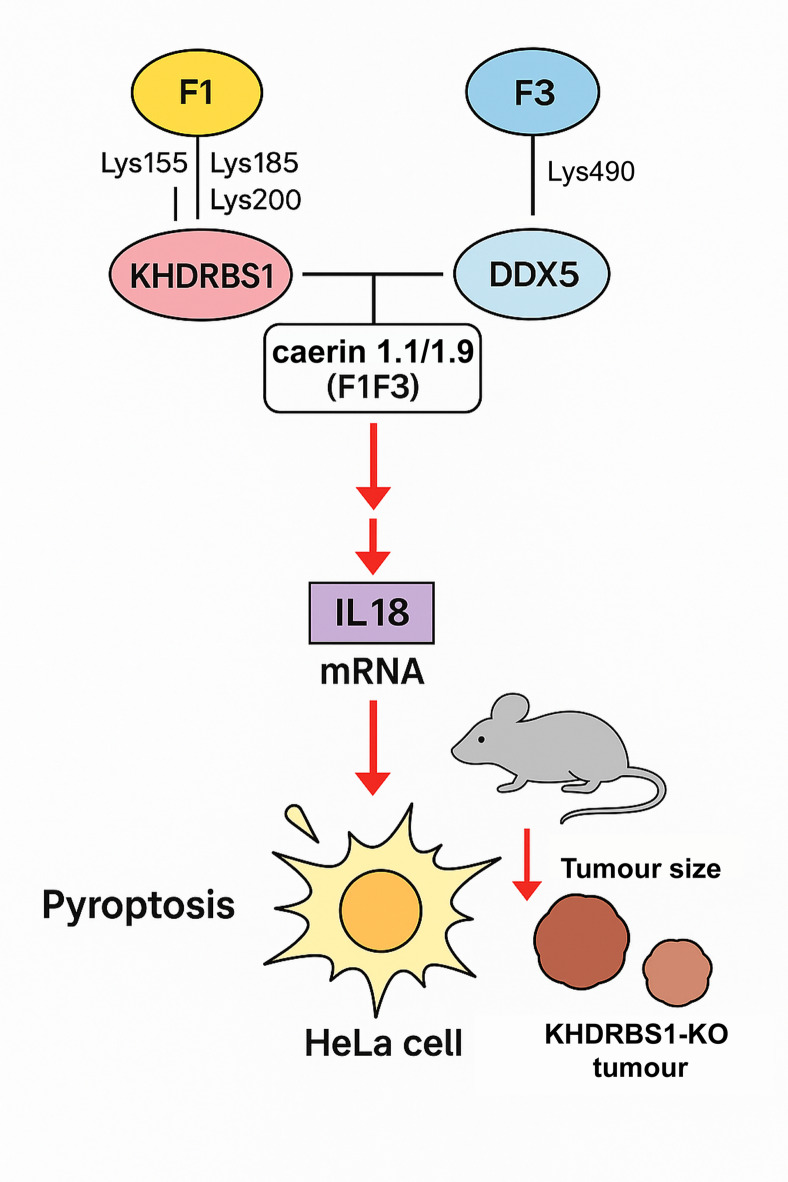
Schematic illustration of the molecular mechanism by which F1F3 induces pyroptosis via KHDRBS1 and DDX5 modulation in HeLa tumour-bearing mice. This diagram summarises the proposed mechanism wherein F1F3 interact directly with KHDRBS1 and DDX5 proteins within HeLa cells, leading to increased IL-18 secretion and GSDME-independent pyroptosis. The upregulation of IL-18 promotes inflammatory macrophage polarisation and enhances anti-tumour immune responses in a xenograft mouse model. Symbols denote activation (arrows) and interaction (lines); the lower section depicts enhanced tumour regression in KHDRBS1-knockout or DDX5-inhibited HeLa xenografts treated with F1F3.

Additionally, KHDRBS1 and DDX5 are known to physically interact, often in an RNA-dependent manner^57–59^. They co-regulate alternative splicing of oncogenes and tumour suppressor genes. This interaction reflects their coordinated role in RNA metabolism and gene expression control, particularly under stress or stimulation conditions. The interaction of F1 and F3 with KHDRBS1 and DDX5, respectively, suggests a novel mechanism by which these host-defence peptides modulate cellular immune responses and cell death pathways in HeLa cells (Fig. 7). F1 interacting to KHDRBS1 at multiple lysine residues (Lys155, Lys185, Lys200) may disrupt its normal role in RNA metabolism, including the regulation of alternative splicing and mRNA translation of immune-related genes. Similarly, F3 interaction with DDX5 at Lys490 may interfere with its helicase activity and RNA processing functions. Together, these interactions appear to compromise the KHDRBS1-DDX5 regulatory axis, thereby enhancing the expression of IL-18 secretion and promoting pyroptotic signalling. This disruption leads to enhanced immune activation and tumour cell susceptibility to peptide-induced cytotoxicity, highlighting a potential therapeutic avenue to augment antitumour immunity by targeting RNA-binding proteins.

In vivo validation in NSG mice, which lack T, B, and NK cells, further emphasized the duality of KHDRBS1’s role. While wild-type HeLa tumours failed to respond to F1F3 in immunodeficient NSG hosts, consistent with previous data in TC-1 nude mice^63^ KHDRBS1 KO HeLa tumours remained sensitive to F1F3 despite the absence of adaptive immunity (Fig. 5C). Notably, KHDRBS1 KO HeLa tumours exhibited accelerated growth in NSG mice, suggesting a potential tumour suppressor role of KHDRBS1 in immunodeficient contexts. This finding contrasts with clinical data linking KHDRBS1 overexpression to poor prognosis in various cancers, including cervical, breast, and prostate malignancies^24,26^. These results imply that KHDRBS1 may exert context-dependent functions, acting as either a tumour promoter or suppressor depending on immune context and tumour lineage, reinforcing the need for precision in its therapeutic targeting.

Moreover, both wild-type and KHDRBS1 KO HeLa tumours responded to F1F3 with increased immune cell infiltration. Flow cytometry and multiplex immunofluorescence analyses revealed elevated CD45^+^ leukocytes, macrophages, and neutrophils in both tumours and spleens following F1F3 administration (Fig. 6A–D). Histological and immunofluorescence analysis further confirmed an increase in iNOS^+^CD86^+^ M1-like macrophages and a corresponding decrease in CD206^+^CD163^+^ M2-like macrophages (Fig. 6E,G), in line with our previous observations in TC-1 and B16 models^35^. These findings support the notion that F1F3 reprograms macrophage polarization toward an antitumour M1 phenotype, contributing to its immunomodulatory and therapeutic potential.

## Conclusion

In summary, our study identifies KHDRBS1 as a critical regulatory node in the cellular response to F1F3 polypeptide treatment. We demonstrate that F1 interacts with KHDRBS1, and that genetic ablation of KHDRBS1 sensitizes HeLa cells to F1F3-induced cytotoxicity. Notably, KHDRBS1 knockout significantly enhances IL-18 secretion in response to F1F3, whereas overexpression of KHDRBS1 in wild-type HeLa cells markedly suppresses this cytokine release, establishing KHDRBS1 as a negative regulator of IL-18 under F1F3-induced stress. Paradoxically, in vivo xenograft experiments revealed that KHDRBS1-deficient tumours grew more rapidly than their wild-type counterparts, suggesting a context-dependent, potentially tumour-suppressive role of KHDRBS1 in this model. This dual functionality in attenuating inflammatory cytokine release while modulating tumorigenicity highlights the complex and multifaceted role of KHDRBS1 in both immunoregulation and cancer biology. Collectively, these findings provide new mechanistic insights into F1F3-mediated immune modulation and underscore the importance of KHDRBS1 as a candidate target for therapeutic intervention. Further investigation is warranted to elucidate the precise signaling pathways by which KHDRBS1 orchestrates the balance between immune activation and tumour progression.

## Electronic supplementary material

Below is the link to the electronic supplementary material.

Supplementary Material 1

## Data Availability

Data is provided within the manuscript or supplementary information files.
