# Supplementary material for: Caerin 1.1/1.9 interfere KHDRBS1-DDX5 regulatory axis to induce IL-18 mediated pyroptosis in a HeLa cell tumour model
Source: Sci Rep. 2025 Jul 30;15:27866. doi: 10.1038/s41598-025-12450-4 (PMC12311210; doi:10.1038/s41598-025-12450-4)
Supplement: Supplementary file 1 — Supplementary Material 1 [file 41598_2025_12450_MOESM1_ESM.pdf]

Supplementary Information

Caerin 1.1/1.9 interfere KHDRBS1-DDX5 regulatory axis to induce IL-18 mediated pyroptosis in a HeLa cell tumour model

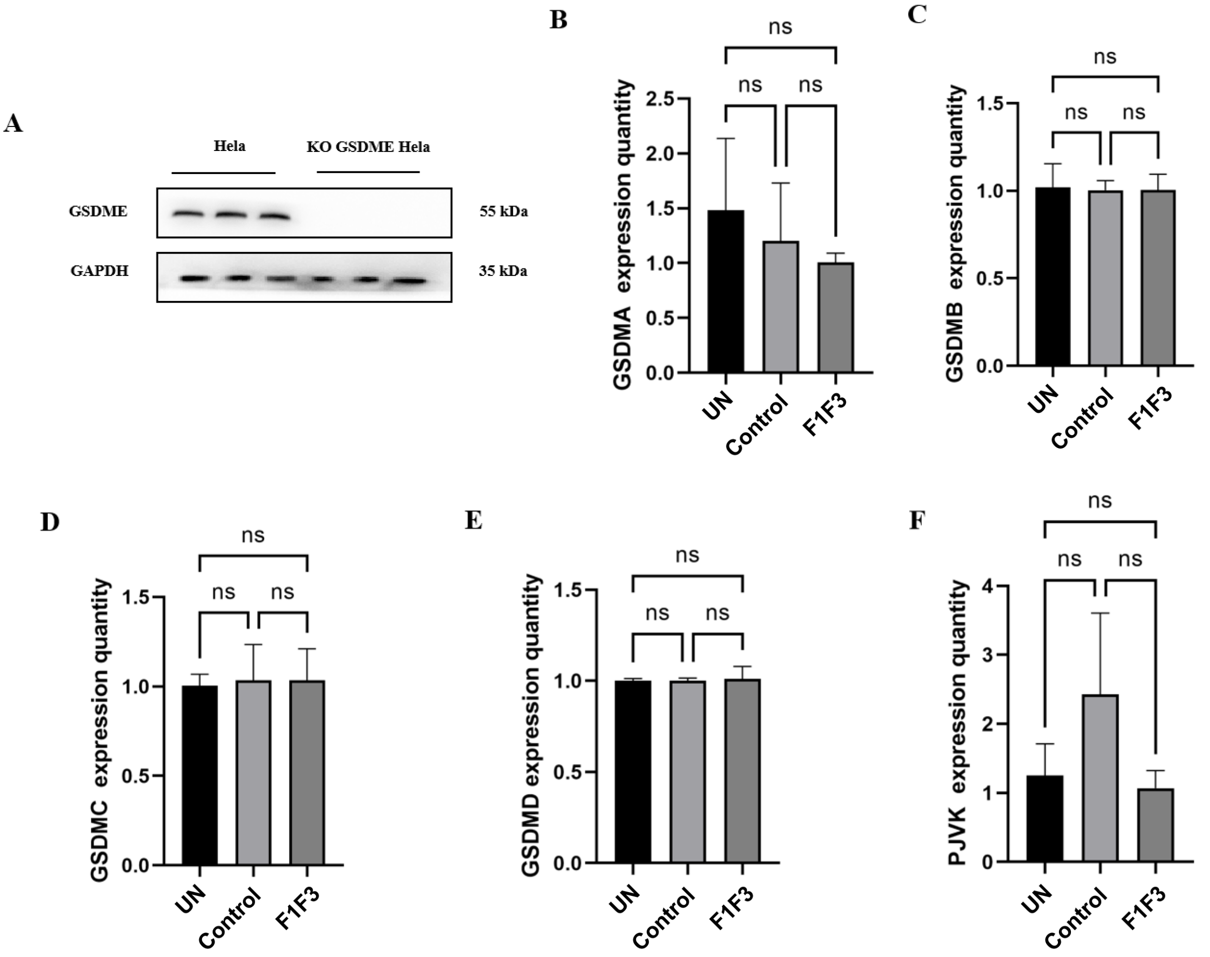

Figure S1 Generation of GSDME-knockout HeLa cells and analysis of gasdermin family gene expression following F1F3 stimulation. (**A**) GSDME-knockout (KO) HeLa cells were generated using CRISPR-Cas9 gene editing, and the absence of GSDME protein expression was confirmed by Western blot analysis. (**B**–**F**) The expression levels of other gasdermin family members were examined by qPCR following 1-hour stimulation with F1F3. No significant changes were observed in the transcriptional levels of GSDMA (**B**), GSDMB (**C**), GSDMC (**D**), GSDMD (**E**), or PJVK (**F**), indicating that F1F3 does not transcriptionally upregulate alternative gasdermin isoforms under these conditions.

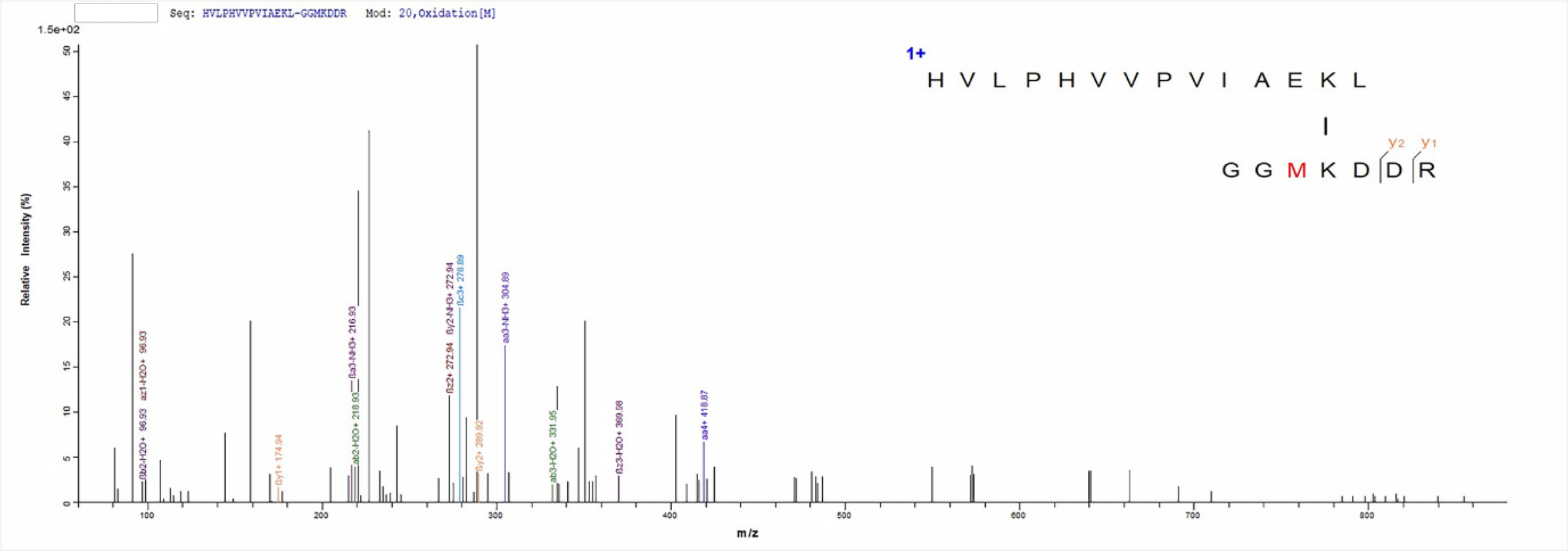

Figure S2 MS/MS spectra showing F3–DDX5 interaction identified by cross-linking mass spectrometry. MS/MS spectra revealed specific crosslinks between F3 and Lys490 on DDX5.

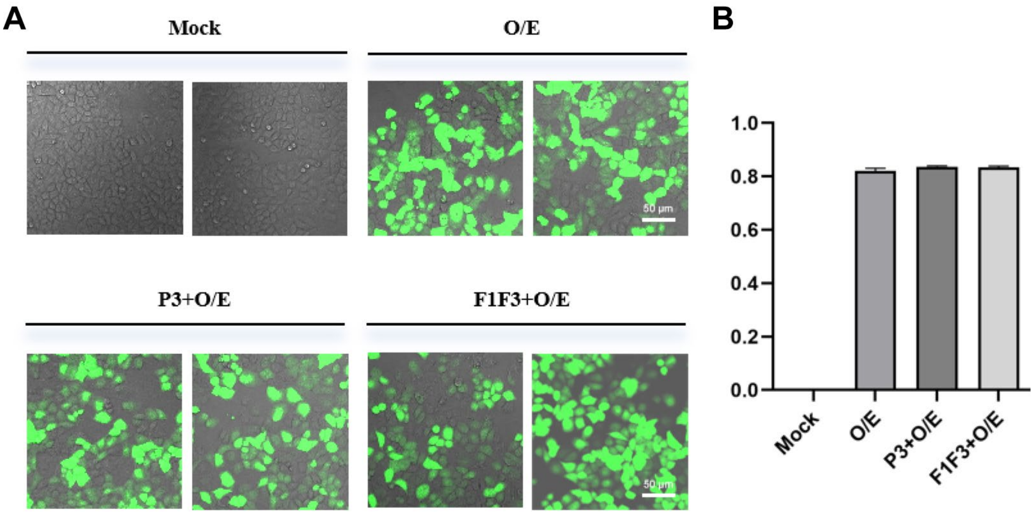

Figure S3 Transfection and quantification of transfection efficiency. (**A**) Transfection of HeLa cells was verified using live-cell imaging on a laser scanning confocal microscope (Zeiss LSM 880) equipped with environmental control settings to maintain optimal temperature and CO_2 levels. Cells were maintained in PBS during imaging to preserve viability and minimise background fluorescence. (**B**) Transfection efficiency was quantified by calculating the proportion of fluorescently labelled cells relative to the total number of cells in the blank (non-transfected) control group.

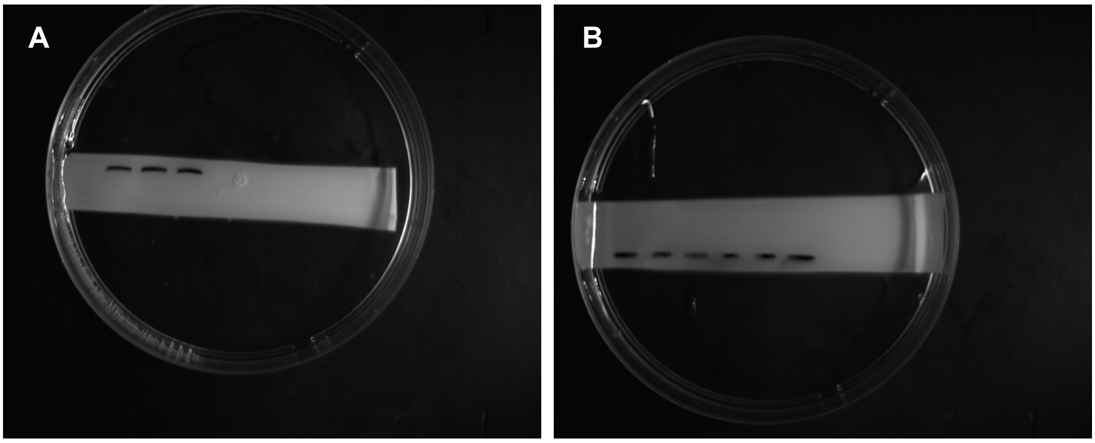

Figure S4 Original Western blot images validating GSDME knockout in HeLa cells. (**A**) Western blot showing the absence of GSDME protein expression in GSDME-KO HeLa cells, corresponding to **Figure S1A**. (**B**) Western blot showing GAPDH expression as a loading control in the same samples.

Cell Use Instruction

DDX5 knockout cell line (Hela)

Cell Info

Catalog	YKO-HS10375		
KO Cell Name	DDX5 knockout cell line (Hela)	Morphology	Epithelial-like, adherent
Gene Name	DDX5	Passage ratio	1:2~1:4
Culture method	90%DMEM+10% FBS;		
	Ubigene didn't use P/S. But client could use P/S after cells grow in good condition after thawing.		
Cryopreservation solution	50%DMEM+40%FBS+10%DMSO		
Special Note	None		

Cell Line Validation Data

Compound heterozygous#B7 :

Allele 1: knock in 1 bp (E2 is knock in 1 bp)

AATCTTGATGAGCTGCCTAAATTTGAGAAGAATTTTTATCAAGAGCACCCTGA(T)TTTGGCTAGGCGCACAGCA

Allele 2: knock in 2 bp (E2 is knock in 2 bp)

ATCTTGATGAGCTGCCTAAATTTGAGAAGAATTTTTATCAAGAGCACCCTGA(TT)TTTGGCTAGGCGCACAGCA

Allele 2: del 1 bp (E2 is deleted 1 bp)

AATCTTGATGAGCTGCCTAAATTTGAGAAGAATTTTTATCAAGAGCACCCTGA~~(T)~~TTTGGCTAGGCGCACAGCA

Note: Upper letters refer to the exon sequence while lower letters refer to the intron sequence.

Red letters (crossed) refer to the deleted sequence.

KO Cell image

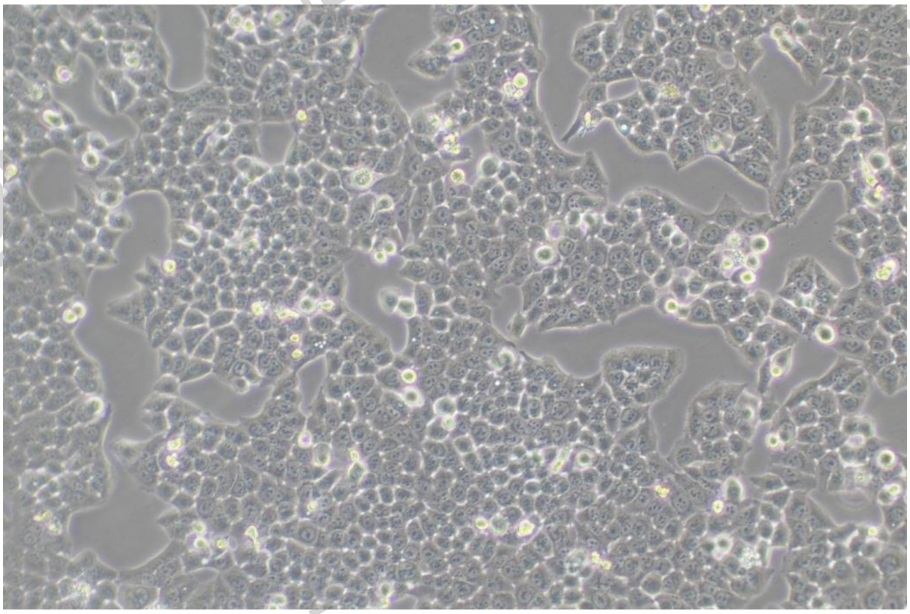

Cell Reception

Cryopreserved cells:

In the case of cryopreserved cells transported with dry ice, upon received, immediately transfer to liquid nitrogen for storage or store briefly at -80°C freezer, or proceed directly to cell thawing. Upon cell thawing, please count the cell number and cell viability and take some photos of the cells under different

magnification (e.g. at 100x and 40x) as the records.

Notice: Upon received, please ensure to takes photos of the package, including dry ice and the tubes, and contact us within 24 hrs if any abnormalities such as dry ice has ran out, the cap of the cryovial is dislodged, broken and the cell is contaminated.

Cell Thawing

1) Preparation: warm up the complete culture medium in 37°C water bath for 30 mins. Transfer the cryopreserved vial from liquid nitrogen to - 80°C freezer, and leave for several minutes to volatilize residual liquid nitrogen;
2) Inside the ultra-clean bench, pipet 6-7 ml of complete medium into a 15 ml centrifuge tube;
3) Take out the cryopreserved vial from - 80℃ freezer and leave in dry ice temporarily, shake slightly before thawing to remove residual dry ice and liquid nitrogen. Then hold the cap with forceps, quickly thaw cells in a 37°C water bath by gently swirling the vial (Note: keep the cap out of the water). In about 1 minute, it would completely thaw;
4) Inside the ultra-clean bench, sterilize the outer surface of the vial by wiping with an alcohol cotton pellet and leave it to dry. Transfer the thawed cells to the prepared centrifuge tube (step 2) by pipette, close the lid, and centrifuge at 1100 rpm for 4 mins at room temp to collect the cells;
5) Inside the ultra-clean bench, carefully remove and discard the supernatant. Resuspend cell pellet with 1ml of fresh complete medium and then transfer to a T25 flask containing 4 ml of complete medium, label the flask with cell name, date and passage no., incubate the flask in a 37℃, 5%CO_2 incubator.

Note: Please do not thaw the cells directly to a T75 flask or 10 cm culture dish.

Cell Passaging

1) As long as the cells are 80%-90% confluent, it is ready to passage. Inside the ultra-clean bench, remove and discard the medium from the flask and briefly rinse the cell 1-2 times with 1×PBS (2-3 ml for T25 flask, 4-5 ml for T75) to remove residual medium and serum;
2) Add the corresponding volume of trypsin solution (see below table 1 for details) and allow trypsin completely cover the cells, place the flask into the incubator and incubate for 1-2 mins (If cells are hard to digest, allow appropriate extension of incubation), until the majority of the cells become round and non-adherent as observed under the microscope, a large number of cells detached from each side when gently shaking and tapping the flask, terminate trypsin digestion immediately;
3) Add complete medium to stop digestion, the volume is 2 times of trypsin. Then gently pipet the cells several times to allow all cells to be completely detached from the flask;
4) Transfer the cell suspension with a 10 ml pipette into a 50 ml centrifuge tube, rinse the residual cells from the flask using appropriate volume of PBS , then collect and put them together to the centrifuge tube;
5) Centrifuge at 1100 rpm for 4 mins at room temp. After centrifugation, remove and discard the supernatant and resuspend the cells with 2 ml of complete medium;
6) Cells need to be passaged at appropriate passage ratio, 1:3 for the first passage, increasing the passaging ratio if the cells are grown to confluence within two days, or decreasing the passaging ratio if the cells are not grown to confluence in 3-4 days.

Table 1. Volume of Trypsin solution added to different size of culture plates/flasks

Size of culture plates/flasks	Trypsin Volume added
6-well plate	0.5mL

T25	1mL
T75	2-3mL
T175	3-4mL

Cell cryopreservation

1) Same as procedures of cell passaging, inside the ultra-clean bench, digest the cells to a single-cell suspension, and terminate digestion by adding complete medium. All liquid is transferred to a 50 ml centrifuge tube;
2) Mix well by pipetting and take 20 μL for cell counting;
3) Centrifuge at 1100 rpm for 4 mins at room temp. After centrifugation, remove and discard the supernatant, and resuspend the cells with 1-2 ml of 4℃ pre-cooled cryopreservation medium (use the one you usually use in lab, or any commercial cryopreservation solutions are fine), then add cryopreservation medium to adjust to the required density (1M cells/ml);
4) Aliquot the cell suspension to cryovials as 1 ml/tube, close the lid tightly, and the cryovials should be labeled with the cell name, source, cell passage number, and date of cryopreservation in advance;
5) Place the cryovials in 4°C pre-cooled Freezing Container, then put the container in -80℃ freezers;
6) Stay overnight, transfer the cryovials to liquid nitrogen for long-term storage.
